# Supplementary material for: Overview of Prognostic Systems for Hepatocellular Carcinoma and ITA.LI.CA External Validation of MESH and CNLC Classifications
Source: Cancers (Basel). 2021 Apr 2;13(7):1673. doi: 10.3390/cancers13071673 (PMC8037197; doi:10.3390/cancers13071673)
Supplement: Supplementary file 1 [file cancers-13-01673-s001.pdf]

cancers

MDPI

Review

Overview of Prognostic Systems for Hepatocellular Carcinoma and ITA.LI.CA External Validation of MESH and CNLC Classifications

Alessandro Vitale [1], Fabio Farinati [1], Michele Finotti [1,*], Chiara Di Renzo [1], Giuseppina Brancaccio [2], Fabio Piscaglia [3,4], Giuseppe Cabibbo [5] Eugenio Caturelli [6], Gabriele Missale [7], Fabio Marra [8], Rodolfo Sacco [9], Edoardo G Giannini [10], Franco Trevisani [11,*], Umberto Cillo [1], Associazione Italiana per lo Studio del Fegato (AISF) HCC Special Interest Group [12,†] and Italian Liver Cancer (ITA.LI.CA) Study Group[11 ‡]

1 Department of Surgery, Oncology and Gastroenterology, University of Padua, 35121 Padua, Italy; alessandro.vitale.10@gmail.com (A.V.); fabio.farinati@unipd.it (F.F.); mi6le@libero.it (M.F.); germanclamp@outlook.com (C.D.R.); umberto.cillo@gmail.com (U.C.)
2 Infectious Diseases Unit, Department of Internal Medicine, Padua University Hospital,35123 Padua, Italy; ggbrancaccio@gmail.com
3 Division of Internal Medicine, Hepatobiliary and Immunoallergic Diseases, IRCCS Azienda Ospedaliero-Universitaria di Bologna, 40138 Bologna, Italy; fabio.piscaglia@unibo.it
4 Department of Medical and Surgical Sciences, Alma Mater Studiorum, University of Bologna, 40138 Bologna, Italy; fabio.piscaglia@unibo.it
5 Department of Health Promotion, Mother & Child Care, Internal Medicine & Medical Specialties, PROMISE, Gastroenterology & Hepatology Unit, University of Palermo, 90127 Palermo, Italy; g.cab@libero.it
6 Gastroenterology Unit, Belcolle Hospital, 01100, Viterbo, Italy; e.caturelli@tiscalinet.it
7 Infectious Diseases and Hepatology Unit, Azienda Ospedaliero-Universitaria of Parma, 43126, Parma, Italy; gabriele.missale@unipr.it
8 Department of Experimental and Clinical Medicine, Internal Medicine and Hepatology Unit, University of Firenze, 50139 Firenze, Italy; fabio.marra@unifi.it
9 Gastroenterology and Digestive Endoscopy Unit, Foggia University Hospital, 71122 Foggia, Italy; saccorodolfo@hotmail.com
10 Gastroenterology Unit, Department of Internal Medicine, University of Genoa, IRCCS Ospedale Policlinico San Martino, 16132, Genoa, Italy; egiannini@unige.it
11 Division of Semeiotics, IRCCS Azienda Ospedaliero-Universitaria di Bologna, 40138 Bologna, Italy, franco.trevisani@unibo.it
12 HCC Special Interest Group, Associazione Italiana per lo Studio del Fegato (AISF), 00199 Roma, Italy
* Correspondence: mi6le@libero.it (M.F.); franco.trevisani@unibo.it (F.T.)
† Membership of the AISF HCC Special Interest Group is provided in the Acknowledgments.
‡ Membership of the Italian Liver Cancer (ITA.LI.CA) study group is provided in the Acknowledgments.

Citation: Vitale, A.; Farinati, F.; Finotti, M.; Renzo, C.D.; Brancaccio, G.; Piscaglia, F.; Cabibbo, G.; Caturelli, E.; Missale, G.; Marra, F.; et al. Overview of Prognostic Systems for Hepatocellular Carcinoma and ITA.LI.CA External Validation of MESH and CNLC Classifications. *Cancers* **2021**, *13*, 1673. https://doi.org/10.3390/cancers13071673

Academic Editor: Alfred Sze-Lok Cheng

Received: 28 February 2021
Accepted: 25 March 2021
Published: 2 April 2021

Publisher's Note: MDPI stays neutral with regard to jurisdictional claims in published maps and institutional affiliations.

Copyright: © 2021 by the authors. Licensee MDPI, Basel, Switzerland. This article is an open access article distributed under the terms and conditions of the Creative Commons Attribution (CC BY) license (http://creativecommons.org/licenses/by/4.0/).

Table S1. Patient Characteristics in the Study Group.

Variables	*n* = 6,882<br>Number of patients (%)<br>Median (IQR)
Age (years)	69 (60-75)
Female sex	1581 (22.97)
HCV	3625 (57.34)
HBV	857 (14.42)
Alcohol	2589 (43.47)
MAFLD	1155 (16.78)
Performance status>0	1949 (28.32)
CRPH	5501 (79.93)
Child Classes B-C	2140 (31.10)
MELD score	10 (8-12)
Diameter of the largest nodule (cm)	3 (2-5)
Multinodular HCC	3254 (47.28)
Macroscopic vascular invasion	972 (14.12)
Metastases	314 (4.56)
Main therapy	
LT	300 (4.36)
LR	1038 (15.08)
ABL	2069 (30.06)
IAT	1549 (22.51)
Systemic	402 (584)
Other	725 (10.54)
BSC	799 (11.61)

Abbreviations: IQR, interquartile range; HCV, hepatitis C virus; HBV, hepatitis B virus; MAFLD, metabolic associated fatty liver disease; CRPH, clinically relevant portal hypertension; MELD, model for end stage liver disease; LT, liver transplantation; LR, liver resection; ABL, ablation; IAT, intra-arterial therapy; BSC, best supportive care.

Table S2. Discrimination ability of different HCC prognostic systems. Distribution of patients in different points/stages of the prognostic systems and corresponding observed median survivals. Log-rank test resulted P<0.0001 for all prognostic systems.

Score/system	Number of patients (%)	Observed median survival (months)	Lower 95%	Higher 95%
All	6,882	32	31	34
ITA.LI.CA score				
0	470 (6.8)	93	81	115
1	1075 (15.6)	65	60	71
2	1317 (19.1)	47	44	52
3	1115 (16.2)	35	31	37
4	720 (10.5)	26	23	29
5	625 (9.1)	20	17	22
6	497 (7.2)	14	12	16
7	386 (5.6)	10	9	14
8	303 (4.4)	7	6	9
9-13	374 (5.4)	4	4	5
ITA.LI.CA staging				
0	1146 (16.7)	61	57	65
A	1658 (24.1)	55	51	59
B1	1625 (23.6)	34	31	36
B2	539 (7.8)	23	21	27
B3	472 (6.9)	14	12	15
C	575 (8.4)	11	9	12

D	867 (12.6)	8	7	10
MESH score				
0	686 (10.0)	79	70	88
1	1656 (24.1)	52	48	55
2	1757 (25.5)	36	33	38
3	1323 (19.2)	24	22	26
4	941 (13.7)	14	12	16
5	419 (6.1)	6	5	7
6	100 (1.5)	3	2	4
CNLC staging				
Ia	2900 (42.1)	56	53	59
Ib	1087 (15.8)	36	32	40
IIa	828 (12.0)	28	26	31
IIb	622 (9.0)	18	17	21
IIIa	697 (10.1)	9	8	11
IIIb	252 (3.7)	11	8	13
IV	496 (7.2)	7	6	9
BCLC classification				
0	1188 (17.3)	59	56	64
A	2731 (39.7)	46	45	49
B	1099 (16.0)	27	25	30
C	1368 (19.9)	12	10	13
D	496 (7.2)	7	6	9
CLIP score				
0	4254 (61.8)	46	44	49
1	1294 (18.8)	30	27	32
2	737 (10.7)	16	14	18
3	342 (5.0)	8	7	10
4-6	255 (3.7)	4	4	5

Abbreviations: ITA.LI.CA, Italian Liver Cancer; CNLC, Chinese Liver Cancer; BCLC, Barcelona Clinic Liver Cancer; CLIP, Cancer Liver Italian Program.
